# A nonlinear and time-dependent leak current in the presence of calcium fluoride patch-clamp seal enhancer

**DOI:** 10.12688/wellcomeopenres.15968.2

**Published:** 2021-11-02

**Authors:** Chon Lok Lei, Alan Fabbri, Dominic G. Whittaker, Michael Clerx, Monique J. Windley, Adam P. Hill, Gary R. Mirams, Teun P. de Boer

**Affiliations:** 1 Institute of Translational Medicine, Faculty of Health Sciences, University of Macau, Macau, China; 2 Department of Biomedical Sciences, Faculty of Health Sciences, University of Macau, Macau, China; 3 Department of Computer Science, University of Oxford, Oxford, Oxfordshire, OX1 3QD, UK; 4 Department of Medical Physiology, Division of Heart and Lungs, University Medical Centre Utrecht, Utrecht, 3584 CX, The Netherlands; 5 Centre for Mathematical Medicine & Biology, School of Mathematical Sciences, University of Nottingham, Nottingham, Nottinghamshire, NG7 2RD, UK; 6 Molecular Cardiology & Biophysics Division, Victor Chang Cardiac Research Institute, Darlinghurst, New South Wales, 2010, Australia; 7 St Vincent's Clinical School, UNSW Sydney, Darlinghurst, New South Wales, 2010, Australia

**Keywords:** electrophysiology, leak current, automated patch, patch clamp, seal enhancer

## Abstract

Automated patch-clamp platforms are widely used and vital tools in both academia and industry to enable high-throughput studies such as drug screening. A leak current to ground occurs whenever the seal between a pipette and cell (or internal solution and cell in high-throughput machines) is not perfectly insulated from the bath (extracellular) solution. Over 1 GΩ seal resistance between pipette and bath solutions is commonly used as a quality standard for manual patch work. With automated platforms it can be difficult to obtain such a high seal resistance between the intra- and extra-cellular solutions. One suggested method to alleviate this problem is using an F ^−^ containing internal solution together with a Ca ^2+^ containing external solution — so that a CaF _2_ crystal forms when the two solutions meet which ‘plugs the holes’ to enhance the seal resistance. However, we observed an unexpected nonlinear-in-voltage and time-dependent current using these solutions on an automated patch-clamp platform. We performed manual patch-clamp experiments with the automated patch-clamp solutions, but no biological cell, and observed the same nonlinear time-dependent leak current. The current could be completely removed by washing out F ^−^ ions to leave a conventional leak current that was linear and not time-dependent. We therefore conclude fluoride ions interacting with the CaF _2_ crystal are the origin of the nonlinear time-dependent leak current. The consequences of such a nonlinear and time-dependent leak current polluting measurements should be considered carefully if it cannot be isolated and subtracted.

## Introduction

Voltage-clamp and current-clamp configurations of the patch clamp technique have been vital tools for studying electrophysiology since the time of Hodgkin & Huxley (1952). Voltage-clamp experiments are commonly used to study voltage and time dependence of ion currents, while current-clamp experiments are used to study for example action potentials of excitable cells. Many different techniques have been developed and one of most the widely-used methods is whole-cell patch clamping ( Sakmann & Neher, 1984).

Whole-cell patch-clamp experiments can be performed using either manual control of a pipette’s position or on automated high-throughput machines based on pores and microfluidics. Manual patch is the conventional method, but it can be very time consuming and low-throughput; whilst automated platforms allow high-throughput recordings, which can be extremely useful for studies that require high numbers of measurements such as drug screening in the pharmaceutical industry ( Bell & Fermini, 2021; Elkins *et al.*, 2013). In recent years many studies have begun to use automated patch-clamp systems to study ion channel electrophysiology ( Gertler *et al.*, 2019; Kang *et al.*, 2019; Kozek *et al.*, 2020; Lei *et al.*, 2017; Lei *et al.*, 2019a; Lei *et al.*, 2019b; Li *et al.*, 2017; Ng *et al.*, 2020; Toh *et al.*, 2020; Vanoye *et al.*, 2018).

**Figure 1.**
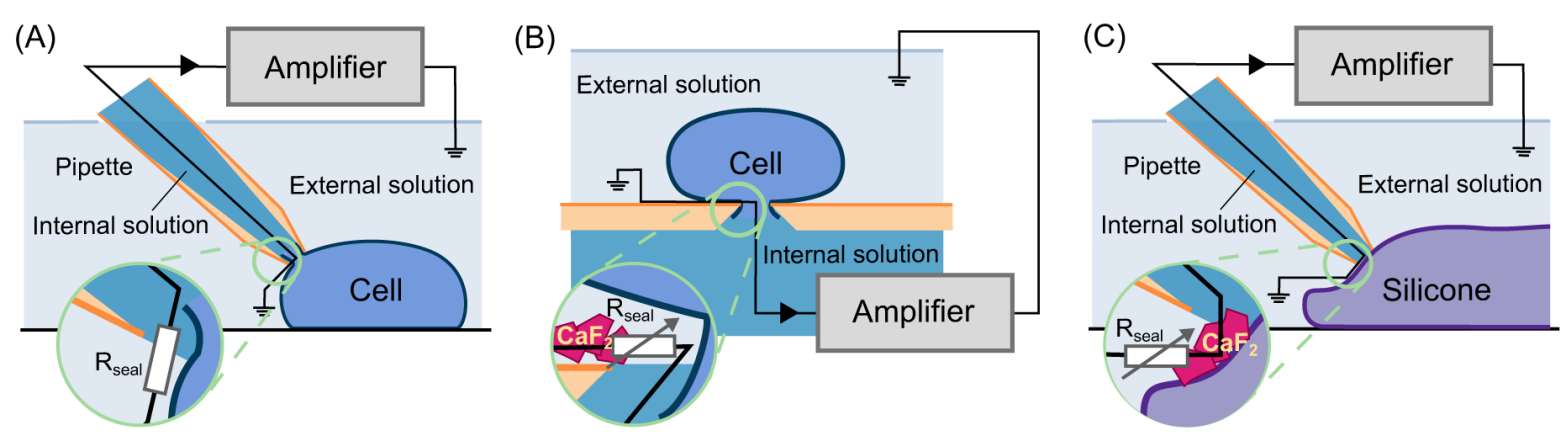
A schematic comparison of manual and automated patch-clamp methods, with a cartoon representation of the leak current circuit. ( **A**) Shows the conventional manual patch-clamp, where a polished glass pipette is used to form a tight electrical seal. ( **B**) Shows the planar design of an automated patch-clamp, where the cell is suspended on top of a micro-pore in the presence of CaF _2_. ( **C**) Shows the set-up of our manual patch-clamp silicone experiments with automated patch-clamp solutions. The magnifications show the difference between the leak current from the three configurations.

A schematic comparison of the two patch-clamp methods is shown in Figure 1A–B. Manual patch-clamp uses a fire-polished glass pipette to form a tight electrical seal (~GΩ) between the pipette tip and the cell membrane. Although the composition of the ionic solutions in the pipette and bath depends on the type of experiments, these solutions are usually intended to be similar to the relevant physiological conditions. Automated platforms, on the other hand, usually have a very different configuration to manual patch, see Figure 1B; they use a design where the cells are suspended on top of a micro-pore on a planar surface. However, this planar design does not always yield as tight a seal (~hundreds of MΩ) as the conventional manual patch-clamp. As a result, some systems recommend the use of seal enhancers that rely on the presence of certain additional ions in the two solutions. For instance, a F ^−^ containing internal solution together with a Ca ^2+^ containing external solution has been used with in both early planar microfluidics setups ( Kostyuk *et al.*, 1975) as well as manual patching ( Maltsev & Undrovinas, 1997; Rugiero *et al.*, 2003; Volkers *et al.*, 2013; Wang *et al.*, 1996; Wendt *et al.*, 1992), and is now employed in many automated platforms (including over 80% of the reported solutions in a recent cross-site and cross-platform comparison of drug screening for a panel of cardiac ion channels, Kramer *et al.*, 2020). The improvement of seal resistance in the presence of these solutions is thought to be due to the formation of CaF _2_ crystals at the interface between the pipette or micro-pore and the cell ( Løjkner *et al.*, 2019), as illustrated schematically in Figure 1B.

Studies have compared manual patch clamping with automated patch clamping data, and showed that their performances are similar ( Billet *et al.*, 2017). Here we examine the reason for some unusual kinetics (dynamics) of a leak current that we observed first on an automated platform, before finding it also appeared in manual patch clamp experiments in the presence of CaF _2_.

**Figure 2.**
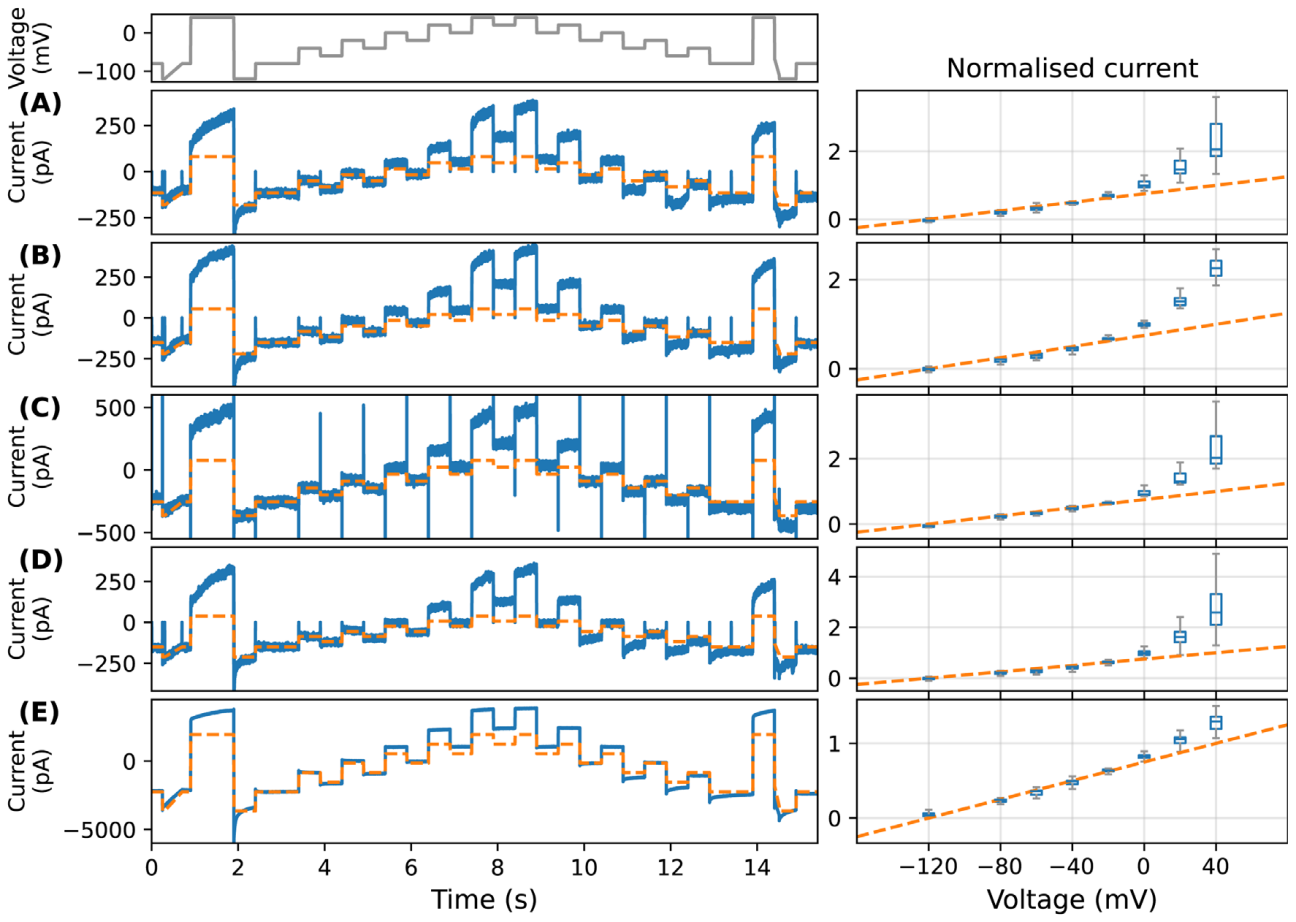
Examples of nonlinear time-dependent leftover current in the presence of CaF _2_. On the left shows typical time series recordings under the staircase protocol (top panel) ( Lei *et al.*, 2019a); right shows the normalized current-voltage (I-V) relationships (at steady state) with a boxplot (range and quartiles) for technical replicates (n). Note that each boxplot can contain more than one data point per cell due to the repeated voltage steps in the protocol. Experimental recordings are shown in blue, and linear leak estimations are shown in dashed orange. The I-V plots are normalized such that the minimum fitted linear leak current (value of the orange line) is 0 and the maximum is 1; note therefore that reversal potential is not at zero on these plots, but this is a simple way to compare results from many cells when leftover current appears to reverse at different voltages. Recordings of the leftover current measured on hERG1a transfected CHO cells after applying an *I*_Kr_-specific blocker (0.5 µM of E-4031) are shown, where experiments were performed at ( **A**) F. Hoffmann-La Roche, Basel ( Lei *et al.*, 2019a; Lei *et al.*, 2019b), n = 15 and ( **B**) an independent reproduction at Victor Chang Cardiac Research Institute in Sydney, n = 10, both using a SyncroPatch 384 automated patch-clamp platform. ( **C** and **D**): Recordings with non-transfected CHO cells, using ( **C**) a manual patch-clamp system (n = 4) and ( **D**) the automated patch-clamp platform (n = 32). ( **E**) Shows a typical recording for an empty well-plate (no biological cells in the solution) in the automated platform (n = 20).

Figure 2A shows an automated patch (Nanion SyncroPatch 384PE) recording of the leftover current measured on Chinese hamster ovary (CHO) cells transfected with the human Ether-à-go-go-Related Gene (hERG)1a after applying a hERG-specific blocker (in this case 0.5 µM of E-4031) at 25°C, with capacitance and 80% series resistance compensations according to manufacturer’s settings. The measurements were done under a voltage-clamp protocol used in Lei *et al.* (2019a), known as the “staircase protocol” (see top panel of Figure 2). One might assume this remaining current consists of both leak current (due to finite resistance of the seal), leftover incompletely blocked hERG, and/or ion currents conducted by non-hERG ion channels natively present in the CHO cells (which we will refer to as ‘endogenous currents’). The measurements (blue) are consistent across laboratories. In Figure 2 we show measurements taken at: (A) F. Hoffmann-La Roche, Basel ( Lei *et al.*, 2019a; Lei *et al.*, 2019b); and (B) Victor Chang Cardiac Research Institute in Sydney (using the same type of Nanion SyncroPatch machine).

**Figure 3.**
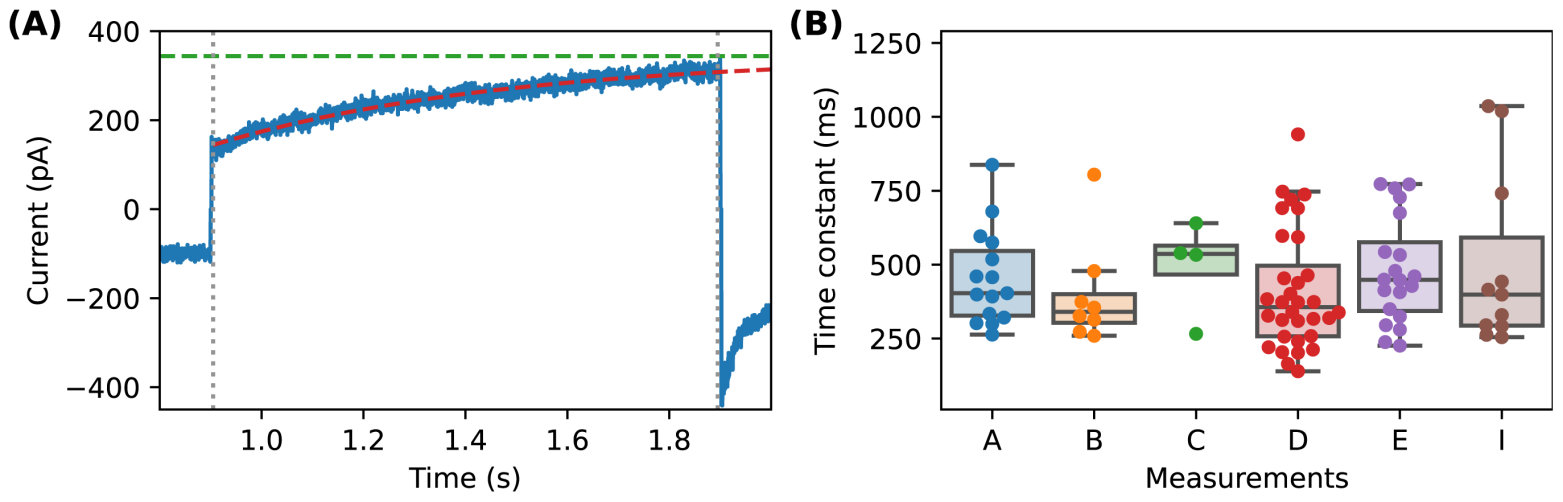
Time constants of the nonlinear time-dependent current in the presence of CaF _2_. ( **A**) Shows an example of the time constant estimation, where the blue line is the data, red is the fitted single exponential curve, the black dotted vertical lines indicate the region of the data used for fitting, and the green horizontal dashed line shows the estimated steady state current, as plotted in Figure 2 I-V plots. ( **B**) Shows the histograms of the estimated time constants from various experiments, with Measurements A-E corresponding to the same set of experiments in Figure 2 and Measurement I the experiments in Figure 4.

The leftover currents are time-dependent when the cell is clamped at a constant voltage; their (normalised) current-voltage (I-V) relationships (at approximately steady state, extrapolated from currents during steps, as described later and in Figure 3A) are non-Ohmic (nonlinear), as shown in Figure 2’s (right panels) blue boxplots for n cells (n = 15 and n = 10). Therefore we refer them to as “nonlinear time-dependent” currents. Measurements were repeated with non-hERG transfected wild-type CHO cells using a manual patch-clamp system (n = 4) and the same automated patch-clamp platform (n = 32), as shown in Figures 2C and 2D, respectively. In the presence of CaF _2_, all measurements exhibit the same nonlinear-in-voltage and time-dependent current. Figure 3 shows the time constants of the current during the first 40 mV step, all measurements shown in Figure 2 have similar distributions of time constants. Initially, these nonlinear currents were thought to be endogenous biological currents, given that they did not take the linear Ohmic form which is usual for leak currents:

$$I_{\text{leak}}=g_{\text{leak}}\times (V_{\text{m}}-E_{\text{leak}}),\;(1)$$

where *V*_m_ is the membrane voltage, and *g*_leak_ and *E*_leak_ are the maximum conductance and reversal potential of the leak current. We illustrate the shape of a linear leak current given by Equation (1) by overlaying a fitted linear current (see Methods section) as an orange dashed line in Figure 2. Note that the leak current here is assumed to be through an imperfect seal, instead of current through some ‘leak channels’ in the cell. In contrast to the commonly-used human embryonic kidney (HEK) cells, CHO cells are thought to have relatively small endogenous currents ( Yu & Kerchner, 1998).

The first question was whether the nonlinear time-dependent leftover current was endogenous current through native ion channels expressed in CHO cells. Figure 2E shows an empty well-plate experiment performed on the automated patch-clamp system (recorded as part of the study by Lei *et al.*, 2019a). That is, the experiments in Figure 2A–B & D were repeated without any biological cells. Since there were no membrane capacitances, no capacitance or series resistance compensations were applied. The recording shows a similar current, except with a much larger amplitude: the linear component appears to dominate – due to an open chip there is very low seal resistance and an enormous (nano- rather than pico-Amperes) linear leak current. But we see the same time constant for the nonlinear time-dependent part of the current ( Figure 3B). This observation suggested the leftover current might not be endogenous CHO cell currents. The question then, is what could cause the nonlinear time-dependent current we observed?

Understanding the origin of the nonlinear time-dependent leftover current is crucial for accurate use of recordings. In the absence of any correction, the nonlinear current will obscure any real ion channel currents. If one uses a linear, non-time dependent leak correction ( Equation (1)), the remaining nonlinear and time dependent current could be mistaken for a real ion channel current, contaminate the recording, and lead to an incorrect characterisation of ion channel gating.

One way to reduce this effect is to use post-blocker subtraction. That is, after measuring the complete current, apply a specific and approximately complete block of the current of interest (e.g. blocking hERG with dofetilide or E-4031) and remeasure the leftover current; the difference between the two recordings should be mainly the current of interest. We used this subtraction method in previous studies where we first observed this non-linear leftover current ( Lei *et al.*, 2019a; Lei *et al.*, 2019b). This should remove the nonlinear time-dependent current from recordings, as well as any other currents that are not specifically blocked. But even then, with post-blocker subtraction the seal resistance could change over time (especially when a relatively long time period is needed for the blocker to have full effect, or when long protocols are required) and the subtraction method would not remove any changes in the nonlinear time-dependent leftover current. Studies without a specific-blocker subtraction method will certainly suffer from polluted currents.

In this study, we examine the origin of the observed nonlinear time-dependent leftover current.

## Methods

The observation of the nonlinear time-dependent current in empty wells ( Figure 2E) suggested this current might not be endogenous, and motivated further investigations using the F ^−^ containing and Ca ^2+^ containing solutions.

Voltage-clamp experiments were performed in the same way as the conventional manual patch experiments except the cell was replaced with a silicone elastomer (‘SYLGARD’), as shown in Figure 1C.

A conventional manual patch-clamp system (HEKA EPC 10 USB Single, HEKA Elektronik GmbH, Lambrecht/Pfalz, Germany) was used for the voltage-clamp experiments. Similar to the no-cell automated experiments, since there was no cell membrane, no capacitance or series resistance compensations were applied. As the pipette tip was gradually moved closer to the silicone elastomer, a seal resistance in the range of 100–1000 MΩ could be obtained, similar to for example Lei *et al.* (2019a); Lei *et al.* (2019b), such that a magnitude of leak current could be measured that was similar to the biological measurements. Leak current between the pipette and the silicone with various patch-clamp solutions was measured and compared to the currents shown in Figure 2A–D.

All codes and data are freely available (see data and software availability Lei & Mirams (2021)).

Note that although we use the seal resistance as a reference of the quality of the seal, we avoid directly quoting the values to avoid overinterpretation. Seal resistance is defined as the inverse of the gradient of the (linear) I-V relationship, thus is usually estimated using two voltage steps in the I-V relationship, ideally when all ion channels are closed. However here we are examining a nonlinear time-dependent leak current, making direct interpretation of the seal resistance values inaccurate.

### Patch-clamp solutions

Three sets of patch-clamp solutions were prepared: (1) a Ca ^2+^ containing external solution; (2) a F ^−^ containing internal solution; (3) a no-F ^−^ internal solution. The concentrations of the solutions are given in Table 1, all substances were obtained from Sigma-Aldrich (Zwijndrecht, The Netherlands). Solutions (1) and (2) are the same as those used in Lei *et al.* (2019a); Lei *et al.* (2019b), which are very similar to other automated patch-clamp studies such as Kozek *et al.* (2020); Ng *et al.* (2020) and those suggested by Nanion for SyncroPatch platforms.

**Table 1.** The patch-clamp solutions used in the experiments. All concentrations are given in mM and product numbers refer to Sigma-Aldrich catalogue. Ca ^2+^ containing (external) solution was titrated to pH 7.4 with HCl; F ^−^ containing internal and no-F ^−^ internal solutions were titrated to pH 7.2 with KOH.

Solutions Product number	NaCl S9625	KCl P4504	KF 449148	MgCl _2_ 63069	CaCl _2_ 21113	HEPES 54457	Glucose G8270	NMDG 66930	Sorbitol S1876	MgATP A9187	EGTA E4387
(1) Ca ^2+^ containing	97.5	4	—	1	**2.05**	10	5	35	20	—	—
(2) F ^−^ containing	10	10	**100**	—	—	10	—	—	—	—	20
(3) No F ^−^	—	130	—	1	—	10	—	—	—	5	5

### Silicone elastomer

The silicone elastomer (SYLGARD 184, The Dow Chemical Company) was prepared using a standard 10:1 ratio of base and catalyst. A thin layer of mixed elastomer was dispensed in 35 mm tissue culture dishes (product number 430165, Corning), and was cured at 60 °C.

### Experimental procedure

Table 2 summarises the three sets of measurements that were performed. The currents were measured under a voltage-clamp protocol used in Lei *et al.* (2019a), known as the “staircase protocol”, shown in Figure 2; a time series file for the protocol is provided (see Data Availability Lei & Mirams (2021)). The holding potential was set to 0 mV. Measurement I is the ‘standard’ configuration using solutions (1) and (2) (the same as in Figure 2), which aimed to reproduce the nonlinear time-dependent leftover current we observed with real cells. Measurement II investigates the current’s dependence on the ionic solutions by swapping the internal and external solutions. Measurement III forms a control experiment by washing out the F ^−^ containing solution in Measurement II with the no-F ^−^ solution; it was performed after Measurement II, such that the F ^−^ containing solution was the external solution and could be easily changed.

**Table 2.** Summary of the three sets of voltage-clamp measurements performed using a manual patch-clamp system with silicone elastomers. Measurement III was performed by washing out the externally applied F ^−^ containing solution in Measurement II with the no-F ^−^ solution, the aim was that the measurements were done in the presence of the CaF _2_ crystal but without F ^−^ in solution.

Measurement	I	II	III
**Internally applied solution**	(2) F ^−^ containing	(1) Ca ^2+^ containing	(1) Ca ^2+^ containing
**Externally applied solution**	(1) Ca ^2+^ containing	(2) F ^−^ containing	(3) No F ^−^

### Data analysis

We estimated *g*_leak_ and *E*_leak_ in Equation (1) using two voltage steps (−80 mV and −40 mV unless otherwise specified); *g*_leak_ was estimated by the ratio of the voltage difference and the mean current difference using the last 500 ms of the voltage steps, after which *E*_leak_ can be directly calculated from Equation (1) using one of the voltage steps (with mean and standard error across all recordings being *E*_leak_ = 8.14 ± 3.38 mV, but we do observe a fair amount of variation in *E*_leak_). The steady state of the nonlinear time-dependent leak current at each voltage step was estimated by fitting a single exponential of the form: *a* × exp(−( *t* − *t*_0_) / τ) + *c*; where *a*, τ, *c* are the parameters to be fitted and *t*_0_ is the starting time of the voltage step. Figure 3A shows an example of such an analysis. The first 5 ms at the beginning of each voltage step was ignored to avoid capacitive spikes. The parameter *c* is then the estimated steady state of the leak current of the given voltage step (as shown in Figure 3A and summarised for many cells in the right column of Figure 2), and the parameter τ is the time constant of the current (as summarised in Figure 3B). For the current-voltage relationships, each current was normalized relative to the linear leak estimate of the current within –120 mV and 40 mV. The analysis was performed in Python using NumPy/SciPy ( Jones *et al.*, 2001); all the code for the analysis is provided (see software availability Lei & Mirams (2021)).

## Results

The empty well-plate measurements in Figure 2E suggested that the nonlinear time-dependent current was non-biological current, and motivated further investigations using the F ^−^ containing and Ca ^2+^ containing solutions. Voltage-clamp experiments were repeated in manual patch with silicone elastomer ( Figure 1C). Using this approach we ensured that: (1) the behaviour of the measured current was not caused by the planar-micro pore configuration ( Figure 1B) or anything specific to the automated platform; and (2) recordings cannot be endogenous biological currents.

### Measurement I

**Figure 4.**
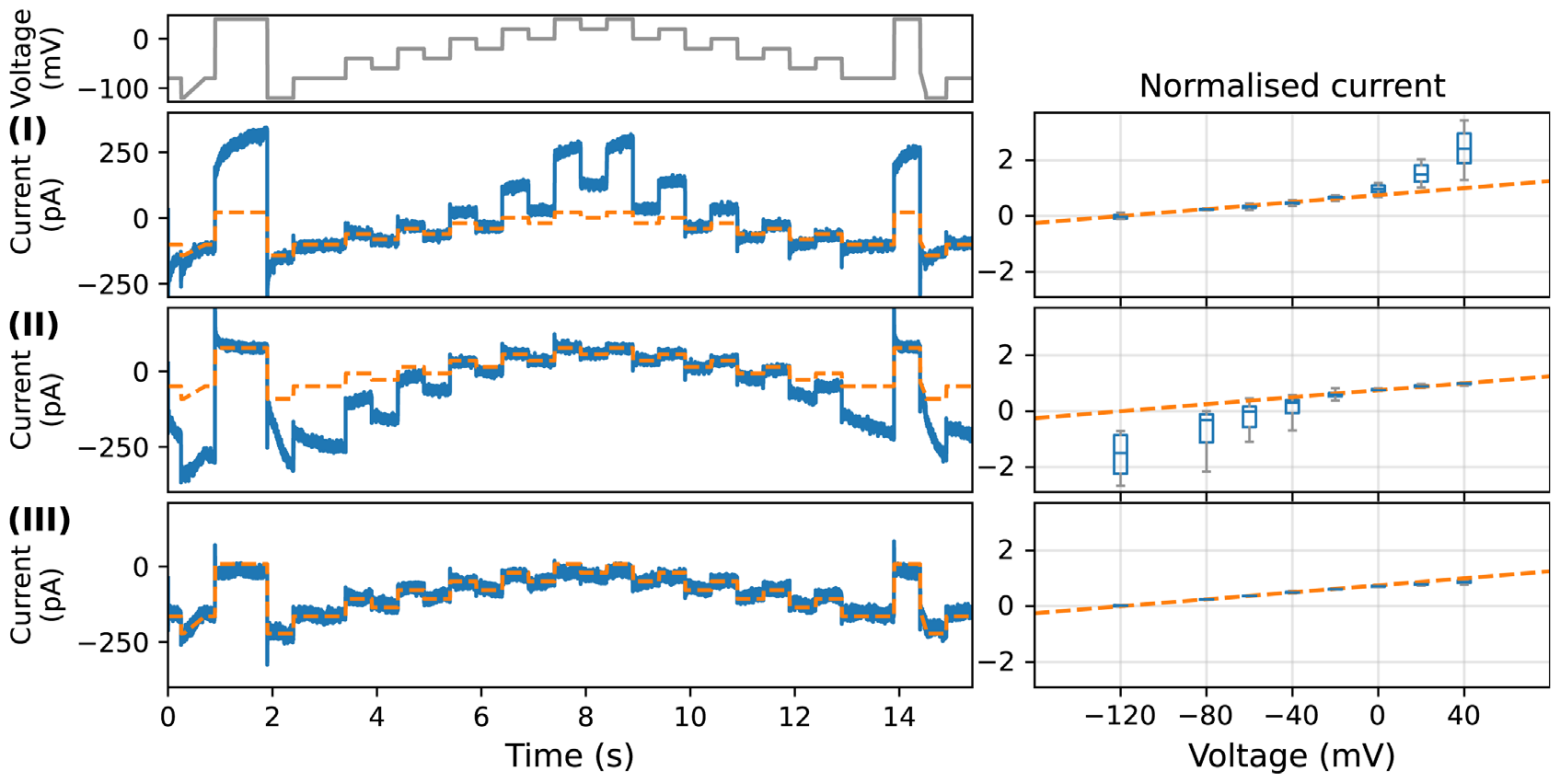
Manual patch-clamp recordings with CaF _2_ solutions on silicone elastomer. Left: typical time series recordings under the staircase protocol (top panel) from Lei *et al.* (2019a); Right: the normalized current-voltage relationships (fitted with a single exponential function, approximating the steady state relation) in the boxplot for n repeats. Experimental recordings are shown in blue, and linear leak estimations are shown in dashed orange. (I)–(III) show the results of Measurements I–III in Table 2. (I) with internal and external solutions containing F ^−^ and Ca ^2+^ respectively (similar to those in Figure 2), with n = 13; (II) with internal and external solutions swapped, the nonlinear portions are now at negative rather than positive voltages (n = 4); (III) same conditions as (II) after F ^−^ was washed out of the bath (n = 4) — the current becomes much closer to the expected linear leak given by Equation (1).

The recorded leak current for Measurement I is shown in Figure 4I, which was measured with the same solutions as in Figure 2 but with silicone elastomer. The leak current measured with silicone elastomer replicates the nonlinear time-dependent current we observed in CHO cells ( Figure 2A–D). Not only were the size of the currents comparable (a few hundred pA), but also the steady state I-V curves were extremely similar to those seen at multiple sites, platforms and cells ( Figure 2A–D). The measured leak current was time-dependent when it was held at a constant voltage, this is most noticeable for the outward (positive) current during an increase of voltage from 0 to +40 mV; the time constants of the current during the first +40 mV step were indistinguishable from those observed in the CHO cell experiments ( Figure 3B). Furthermore, the I-V relationship (at plateau) was non-Ohmic (nonlinear).

### Measurement II

In this set of measurements, we repeated the experiments but swapped the internal and external solutions in Measurement I, to investigate the current’s dependence on the ionic solutions. Figure 4II shows the recorded leak current for Measurement II. Both the time-dependent part of the leak current and its nonlinear I-V relationship were reversed. Instead of a prominent outward (positive) time-dependent current during an increase of voltage from approximately 0 to +40 mV, a noticeable inward (negative) time-dependent current was produced during a decrease of voltage to approximately −80 to −120 mV. Moreover, the nonlinear I-V relationship (at plateau) changed from superlinear in Measurement I to sublinear, as shown in the right panels of Figure 4I–II. Note that, as the nonlinearity was thought to be caused by the inward time-dependent current at low voltage, the linear leak estimation (orange dashed line) in Figure 4II was fitted to two voltage steps at higher voltages (+40 mV and +60 mV).

### Measurement III

Finally, as a control experiment, immediately following Measurement II during the same experiment the (now external) F ^−^ containing solution was washed out and replaced with the no-F ^−^ solution. Note that the measurement was performed by washing the external solution in Measurement II after the CaF _2_ crystal was formed, and therefore the measurement should be in the presence of the CaF _2_ crystal, although in some cases (but not all) the magnitude of the current became larger due to the wash perhaps indicating some loss of crystal and seal resistance. The results are shown in Figure 4III. The nonlinear I-V relationship and time-dependent dynamics of the leak current were almost entirely eliminated; linear leak current that follows Equation (1) was observed by simply removing F ^−^ from the solution.

## Discussion

We had observed a nonlinear and time-dependent current whilst taking recordings from CHO hERG1a cells in the presence of a hERG blocker, on an automated patch-clamp platform in the presence of calcium fluoride containing solutions. In this study, we investigated the origin of this ‘leftover current’ as it is crucial for accurately determining the kinetics of ion channel currents ( Lei *et al.*, 2020). Experiments using a conventional manual patch-clamp setup on a silicone elastomer instead of a biological cell were performed with CaF _2_-containing patch-clamp solutions.

Our results (Measurement I) show that it was possible to replicate the nonlinear time-dependent leftover current ( Figure 2A–B) with manual ‘no cell’ (silicone elastomer) experiments ( Figure 4I). Therefore the current was neither a feature of the automated patch clamp system nor an endogenous current from the (CHO) cells, and is predominantly a calcium-fluoride-dependent leak current through the imperfect seal. We then show that by interchanging the internal and external solutions (Measurement II), the time-dependence was retained but the nonlinear I-V relationship of the current was reversed ( Figure 4I–II). This is evidence that the nonlinear time-dependent part of the leak current is determined by the two ionic solutions used. Finally, in Measurement III ( Figure 4III), the nonlinear I-V relationship and time-dependent dynamics of the leak current were eliminated by washing out the externally applied F ^−^ containing solution from Measurement II and replacing with the no-F ^−^ solution. Observing a linear I-V relationship in Measurement III is indeed consistent with a previous study using Sylguard by Sachs & Qin (1993), whilst they also observed a nonlinear current in the presence of arginine. Note that the measurement was performed by washing the F ^−^ containing solution in Measurement II after the crystal was formed, hence the nonlinear time-dependent leak current occurred as a consequence of the presence of the crystal *and* fluoride. This demonstrates that a requisite for the I-V nonlinearity and time-dependent behaviour of the leak current is the F ^−^ containing internal solution used as part of a seal enhancer in automated patch-clamp systems.

We propose the following tentative hypothesis to explain the observed I-V nonlinearity and time-dependent behaviour of the leak current. Our leading conjecture is that the nonlinear time-dependent leak current is ordinary linear leak through imperfect seal with a conductance that changes nonlinearly and time-dependently with voltage. We speculate that the reason for this ‘extra’ current could be defects in the CaF _2_ crystals: F ^−^ has a higher mobility than Ca ^2+^, so F ^−^ may preferentially move back into the cell from imperfections in the crystal when voltage is high. The extra defects in the crystal that result (sites missing fluoride, Huisinga, 1999) could allow a larger non-selective leak, forming the measured current. This process would reverse when voltage is low, with fluoride returning to ‘plug the holes’ in the crystal and reducing leak conductance, giving the observed asymmetry in leak current. This is a similar concept to e.g. positively charged polyamines causing block of inward rectifier potassium channels at positive potentials ( Fakler *et al.*, 1995). That is, neither fluoride nor the polyamines contribute strongly to a measured current but cause its block. This hypothesis is consistent with the direction of the ‘extra’ current in the leak in both Measurements I and II.

Our findings have implications in methods for measuring and post-processing the recordings. The leak current has a nonlinear I-V relationship and time-dependence when it is held at a constant voltage, it is therefore important to subtract it off from the recordings such that a pure current of interest can be obtained. Due to its nonlinearity and time-dependence, the kinetics of the resulting current of interest (hERG1a current in our examples) could be undesirably affected if leak is not carefully removed: the fluoride-dependent leak current can shift the I-V curve of measured currents, alter observed time constants, etc. Some manual patch clamp findings in the literature may also need to be re-examined in light of these observations.

In addition to the extra nonlinear time-dependent leak current, the use of intracellular fluoride and extracellular calcium as a seal enhancer could also give rise to other problems. Using intracellular fluoride, as a calcium chelator, prevents the use of calcium containing intracellular solutions, and fluoride is also a phosphatase inhibitor ( Guranowski, 1990). Using extracellular calcium at higher concentrations than the physiological range can also alter channel gating ( Ho *et al.*, 1998), at least partly due to membrane charge screening ( McLaughlin *et al.*, 1971).

Since the form of this leak current is, to our knowledge, not very well studied; it is not possible to use the standard methods of estimating linear leak current to perform the correction. The standard methods involve a small leak step (change in voltage) at which the ion channel of interest is (nearly) closed. However, given the nonlinearity in the I-V relationship of this current, a voltage-current estimation across one range or pair of voltages would result in an incorrect estimation of the whole I-V relationship (see for example how the orange dashed lines missed the blue crosses in Figure 2 and Figure 4 right panels), and the time-dependent dynamics would not be captured. The best approach available at present is the widely-used block-and-subtract method, as used in our earlier studies ( Lei *et al.*, 2019a; Lei *et al.*, 2019b). However, the seal can change over time (especially if a relatively long period is allowed for a blocker to take effect). In which case, the subtraction will not remove changes in the nonlinear time-dependent portion of the leak current, resulting in over- or under-subtracted nonlinear leak current. Therefore this study raises concerns about the effects and consequences of this nonlinear time-dependent leak/leftover current.

There are two obvious options to account for this current. Firstly, and ideally, we would completely remove this nonlinear time-dependent leak current by altering the ionic solutions. We have seen that the properties of the current depend on the concentration of F ^−^ in the solutions (and will probably also depend on [Ca ^2+^]). Optimal concentrations for these ions may exist that are high enough to sufficiently enhance the seal, but low enough that the nonlinearity and time-dependence are not evident in recordings. Other salts such as BaF _2_, CaSO _4_, etc. should be tested to see whether they can enhance seals but remove this nonlinear time-dependent leak current, as well as examining their physiological effects on the cells ( Tasaki & Takenaka, 1964). It may also be possible to restore a linear leak current by washing away the F ^−^ ions after establishing a seal, as we did in Measurement III.

Secondly, further studies of this F ^−^-dependent current could allow it to be modelled so well that it could be subtracted from recordings in post-processing in much the same way as the linear leak. But at a minimum this option would involve: a better characterisation of the time and voltage dependence of this leak current; its dependence on the concentration of F ^−^ and/or Ca ^2+^ in the solutions; dependence on the seal resistance; and testing that the current is predictable (and not, for example, a function of unmeasured quantities such as crystal size/thickness/volume). We anticipate that removing the current through alterations to the ionic solutions will be simpler and more reliable.

## Conclusions

We recorded a nonlinear and time-dependent current in the presence of Ca ^2+^ and F ^−^ in patch clamp solutions (intended to form a CaF _2_ seal enhancer). The same nonlinear time-dependent current was observed using a conventional manual patch-clamp setup in close proximity to a silicone elastomer to form a seal between 100 to 1000 MΩ. Therefore the current was determined to be mainly leak current through imperfect seal, and not endogenous biological currents. The nonlinear and time-dependent form of the leak current was caused by the presence of CaF _2_ and could be eliminated by F ^−^ washout after CaF _2_ crystal formation.

## Data availability

### Underlying data

All datasets used in the publication are available at: https://github.com/CardiacModelling/nonlinear-time-dependent-leak.

A permanently archived version is available on Zenodo: https://doi.org/10.5281/zenodo.5571252 Lei & Mirams (2021)

This project contains the following underlying data:

- data/protocol-staircaseramp.csv — a time series trace of the voltage protocol.
- data/silicone and data-rev/silicone — a set of voltage-clamp timeseries data in HEKA format for Measurements I, II and III; as plotted in Figure 4)
- data/cho-cell, data-rev/cho-herg and data-rev/cho-herg-2 — a set of CHO-hERG cell voltage-clamp time-series data taken from ( Lei *et al.*, 2019a), as plotted in Figure 2.
- data-rev/cho-empty and data-rev/cho-empty-auto — a set of untransfected CHO cell voltage-clamp time-series data as plotted in Figure 2.
- data/no-cell and data-rev/no-cell — a set of empty well-plate voltage-clamp time-series data taken from ( Lei *et al.*, 2019a), as plotted in Figure 2.

A description of other files, including python scripts to read and plot these data, is available in the repository Readme file.

## Software availability

Source code is also available from: https://github.com/CardiacModelling/nonlinear-time-dependent-leak and was archived at time of publication: https://doi.org/10.5281/zenodo.5571252 Lei & Mirams (2021)

License: BSD 3-Clause
