# Supplementary material for: A nonlinear and time-dependent leak current in the presence of calcium fluoride patch-clamp seal enhancer
Source: Wellcome Open Res. 2021 Nov 2;5:152. Originally published 2020 Jul 1. [Version 2] doi: 10.12688/wellcomeopenres.15968.2 (PMC8591515; doi:10.12688/wellcomeopenres.15968.2)
Supplement: Peer review history

# Peer review: Reviewer response for version 2

**DOI:** 10.21956/wellcomeopenres.19142.r46894

**Published:** 2022-01-07

**Authors:** Andrew Glazer

**Affiliations:** 1 Vanderbilt Center for Arrhythmia Research and Therapeutics, Department of Medicine, Vanderbilt University Medical Center, Nashville, TN, USA

The authors addressed my comments satisfactorily.

Is the work clearly and accurately presented and does it cite the current literature?

Yes

If applicable, is the statistical analysis and its interpretation appropriate?

Yes

Are all the source data underlying the results available to ensure full reproducibility?

Yes

Is the study design appropriate and is the work technically sound?

Yes

Are the conclusions drawn adequately supported by the results?

Partly

Are sufficient details of methods and analysis provided to allow replication by others?

Yes

Reviewer Expertise:

Automated patch clamping.

I confirm that I have read this submission and believe that I have an appropriate level of expertise to confirm that it is of an acceptable scientific standard.

# Peer review: Reviewer response for version 2

**DOI:** 10.21956/wellcomeopenres.19142.r46896

**Published:** 2021-11-16

**Authors:** Carlos Guillermo Vanoye

**Affiliations:** 1 Department of Pharmacology, Feinberg School of Medicine, Northwestern University, Chicago, IL, USA

The authors addressed my concerns, I have only minor text editing comments:

 In the Abstract line 2, it reads as if a leak occurs when the seal is not perfectly insulated from the bath. Please rewrite to mean that a leak occurs when the seal does not perfectly insulate the bath (extracellular) from the internal solution.

 .Abstract line 14: “non-linear-in-voltage” is confusing, please re-write.

 Page 3, column 2, paragraph 4: Line 5:  “Figure 2’s” should read “Figure 2”. Line 8: use “non-transfected CHO cells” instead of non-hERG-transfected WT CHO cells.

 Page 5, paragraph 1: Line 6:  “endogenous biological currents” should read “endogenous currents”.

 Page 6 column 1, Methods paragraph 1, line 2 “empty wells..” should read “cell-free wells”.  

 Page 7, column 1 paragraph 1, line 1: “with real cells should read “with cells”

 Page 7 column 2, Results paragraph 1: “..empty well-plate..” should read “cell-free well-plate”.

 Page 8, column 2, paragraph 1: “..(CHO) cells.” should read  “..CHO cells.”

 The use of the term “no biological cells” throughout the text is misleading because Model Cells were not used when CHO cells were not recorded. I would suggest to use the terms “cell-free” or “in the absence of cells” instead.

Is the work clearly and accurately presented and does it cite the current literature?

Yes

If applicable, is the statistical analysis and its interpretation appropriate?

No

Are all the source data underlying the results available to ensure full reproducibility?

Yes

Is the study design appropriate and is the work technically sound?

Partly

Are the conclusions drawn adequately supported by the results?

Partly

Are sufficient details of methods and analysis provided to allow replication by others?

Yes

Reviewer Expertise:

Ion channels, electrophysiology, molecular biology, cell physiology.

I confirm that I have read this submission and believe that I have an appropriate level of expertise to confirm that it is of an acceptable scientific standard.

# Peer review: Reviewer response for version 2

**DOI:** 10.21956/wellcomeopenres.19142.r46897

**Published:** 2021-11-15

**Authors:** Gildas Loussouarn

**Affiliations:** 1 CNRS, INSERM,the thorax institute, University of Nantes, Nantes, France

I approve the manuscript but I have still a few minor comments regarding Version 2, essentially text edits.

**Regarding my previous comment**: I agree that in the conditions now described, the time-dependent current can not be due to Rs compensation in Figure 2E (no cells).

 But do the authors have another explanation for the fact that the current does not show any clear time dependence when voltage changes from 0 to +40mV  in the middle of panel (E), but only when voltage changes from -80 mV to +40mV (left and right of panel E)? In other conditions, time dependence is visible in both voltage steps (-80 to +40 mV and 0 to +40mV ).

**Abstract : A leak current to ground occurs whenever the seal between a pipette and cell (or internal solution and cell in high-throughput machines) is not perfectly insulated from the bath (extracellular) solution.**

 Something is wrong with this sentence: I would say "A leak current to ground occurs whenever the seal does not insulate the intra-cellular solution (in the pipette in manual patch or the chip in high-throughput machines) from the extracellular (bath) solution."

**Page 3: Studies have compared manual patch clamping with automated patch clamping data, and showed that their performances are similar (Billet *et al*., 2017).**

 I think this sentence is misleading, 'similar performance'  may me interpreted as the quantity of data rather than quality of data.

**Page 3 : as shown in Figure 2’s (right panels) blue boxplot**

 there are several  's  that are not necessary, or even relevant.

**Page 3: Measurements were repeated with non-hERG transfected wild-type CHO cells using a manual patch-clamp system.**

 I would replace with "non-transfected CHO cells"

**Page 7: Eleak can be directly calculated from Equation (1) using one of the voltage steps (with mean and standard error across all recordings being Eleak = 8.14 ± 3.38 mV.**

 Is this value close to liquid junction potential?

**Page 7: this is most noticeable for the outward (positive) current during an increase of voltage from 0 to +40 mV.**

 Should it be "from -80mV to +40mV"? The time dependence is the most noticeable at this voltage step.

**Figure 4. Right: the normalized current-voltage relationships (fitted with a single exponential function, approximating the steady state relation)**

 Not clear to me: it is not the I/V relationship that has been fitted. Please rephrase.

**Page 8. Moreover, the nonlinear I-V relationship (at plateau) changed from superlinear in Measurement I to sublinear, as shown in the right panels of Figure 4I–II.**

 Is it really "moreover" since it is a direct consequence of the above replacement of the outward current by the inward current?

**Page 8 Our results (Measurement I) show that it was possible to replicate the nonlinear time-dependent leftover current (Figure 2A–B) with manual ‘no cell’ (silicone elastomer) experiments (Figure 4I).**

  manual ‘no cell’ is not clear, please rephrase.

Is the work clearly and accurately presented and does it cite the current literature?

Yes

If applicable, is the statistical analysis and its interpretation appropriate?

Partly

Are all the source data underlying the results available to ensure full reproducibility?

Yes

Is the study design appropriate and is the work technically sound?

Partly

Are the conclusions drawn adequately supported by the results?

Partly

Are sufficient details of methods and analysis provided to allow replication by others?

Yes

Reviewer Expertise:

Electrophysiology, patch-clamp.

I confirm that I have read this submission and believe that I have an appropriate level of expertise to confirm that it is of an acceptable scientific standard.

# Peer review: Reviewer response for version 2

**DOI:** 10.21956/wellcomeopenres.19142.r46895

**Published:** 2021-11-11

**Authors:** Marc Rogers

**Affiliations:** 1 Metrion Biosciences Limited, Cambridge, UK

The additional data (experiments with untransfected cells, N replicates) is helpful, as-is the rewording of several text sections.

 However, I would still like to highlight several places where the focus on automated patch clamp (APC) remains and is misleading or potentially biased:

**Abstract**

 The authors neglect to include new data showing that non-linear currents are observed in untransfected CHO cells under manual patch (e.g after sentence #6 'However,...automated patch clamp platform', or sentence #7 'We performed... leak current'.

**Discussion**

 p2, last sentence could also include the fact that they see a similar non-linear current with cell-based manual patch recordings as well.

 Although minor, I would prefer that the authors consider a final edit to warrant full acceptance of this review and proceed to indexing of a final version.

Is the work clearly and accurately presented and does it cite the current literature?

Yes

If applicable, is the statistical analysis and its interpretation appropriate?

Partly

Are all the source data underlying the results available to ensure full reproducibility?

Yes

Is the study design appropriate and is the work technically sound?

Partly

Are the conclusions drawn adequately supported by the results?

No

Are sufficient details of methods and analysis provided to allow replication by others?

Yes

Reviewer Expertise:

Manual patch clamp, automated patch clamp, drug discovery screening, pharmacology, ion channel biophysics.

I confirm that I have read this submission and believe that I have an appropriate level of expertise to confirm that it is of an acceptable scientific standard.

# Peer review: Reviewer response for version 1

**DOI:** 10.21956/wellcomeopenres.17512.r39340

**Published:** 2020-07-30

**Authors:** Carlos Guillermo Vanoye

**Affiliations:** 1 Department of Pharmacology, Feinberg School of Medicine, Northwestern University, Chicago, IL, USA

In the present manuscript, Lei *et al.* describe a rectifying, time-dependent leak current that is fluoride-dependent. This is well written manuscript with very interesting results. Their observation is important because some high throughput systems require the presence of fluoride to enhance seal formation. And the presence of this current, if not corrected properly, could contaminate ion channel recordings complicating results interpretation.

 Comments:

1. Were the current traces shown in Figs 2 and 3 recorded with capacitance and series resistance compensation turned on? The compensations may affect the shape of the observed current. 
2. The seal resistance values for the recordings shown in Figs. 2 and 3 are not given. Those values would allow the reader to interpret the amplitude of the leak current shown. This information is critical specially when comparing the results in Figure 3.II and Figure 3.III.
3. Each of the I-Vs shown in Figures 2 and 3 appear to be derived from only one recording. Please provide data from multiple wells (Fig 2) and patches (Fig 3) and show means, standard errors (or standard deviations) and statistical significance. The conclusions would be supported by providing I-Vs derived from multiple observations not just from one.
4. The slope of the outward current in Fig.2A and 2B is steeper than the one shown in Fig 2C. It is assumed that no currents are going through the cell membrane on those recordings but this may not be the case. Is the block of hERG1a by E-4031 100%? There could also be chloride channels (Gill *et al*, 2006) ^1^ that may be carry the observed current. The conclusion that there is no current going through ion channels in those recordings would be strengthened if cesium was used instead of potassium (or using non-transfected CHO-K1 cells) and chloride channel blockers were added.
5. Regarding the results shown in Figure 3, the authors should compare their observations to those previously reported by Sachs and Qin (1993) ^2^. The previous results showed ion-selective currents in the absence of CaF _2_ using a similar approach (glass pipette plus Sylgard).
6. Is the linearity of the IV curve shown in Fig. 3.III due to the absence of fluoride or to the large drop in seal resistance? Please provide pre- and post-wash seal resistance values. Also multiple recordings (“patches”) would be recommended.
7. Is the drop in resistance due to the loss of fluoride (and presumably CaF _2_ crystals)? Or to the pipette and Sylgard moving apart during the wash? Performing a wash with a fluoride-containing solution could answer this question.

Is the work clearly and accurately presented and does it cite the current literature?

Yes

If applicable, is the statistical analysis and its interpretation appropriate?

No

Are all the source data underlying the results available to ensure full reproducibility?

Yes

Is the study design appropriate and is the work technically sound?

Partly

Are the conclusions drawn adequately supported by the results?

Partly

Are sufficient details of methods and analysis provided to allow replication by others?

Yes

Reviewer Expertise:

Ion channels, electrophysiology, molecular biology, cell physiology.

I confirm that I have read this submission and believe that I have an appropriate level of expertise to confirm that it is of an acceptable scientific standard, however I have significant reservations, as outlined above.

## References

[1] : Development and validation of HTS flux assay for endogenously expressed chloride channels in a CHO-K1 cell line. *Assay Drug Dev Technol* .2006;4(1) : 10.1089/adt.2006.4.65 65-71 10.1089/adt.2006.4.65 16506890

[2] : Gated, ion-selective channels observed with patch pipettes in the absence of membranes: novel properties of a gigaseal. *Biophysical Journal* .1993;65(3) : 10.1016/S0006-3495(93)81149-4 1101-1107 10.1016/S0006-3495(93)81149-4 PMC12258277694669

# Response

**Published:** 2021-10-26

**Authors:** Gary Mirams

**Affiliations:** University of Nottingham, UK

We thank the reviewer for the insightful and thorough review. This post relates to the changes introduced in Version 2 of the manuscript. The reviewer's comments are in bold and italic, with our point-by-point response below.

***Comments:***

***Were the current traces shown in Figs 2 and 3 recorded with capacitance and series resistance compensation turned on? The compensations may affect the shape of the observed current.***

 Indeed the compensation may affect the shapes of the observed current, but given the size of the current, membrane capacitance, and series resistance, both the time dependence (time constant) and the size of the effect (current size) due to membrane capacitance and series resistance are in a completely different scale (and in fact opposite direction) compared to the nonlinear time-dependent current that was observed. A comparison of the capacitance and series resistance effect is shown in Lei *et al.*, 2020 [1].

 Furthermore, the compensations were turned on for the measurements in Figure 2. This should minimise the changes to the shape of the observed current due to cell membrane capacitance and series resistance. Whilst the compensations were turned off for the measurements in Figure 3 (new Figure 4) as silicone elastomers do not have a membrane capacitance which would not give rise to any time-dependent effect.

 We have added the following text to Introduction:

 “ *[Figure 2A shows an automated patch (Nanion SyncroPatch 384PE) recording of the leftover current measured on Chinese hamster ovary (CHO) cells transfected with the human Ether-à-go-go-Related Gene (hERG)1a after applying a hERG-specific blocker (in this case 0.5 µM of E-4031) at 25°C,] with capacitance and 80% series resistance compensations according to manufacturer’s settings.*”

 “ *[That is, the experiments in Figure 2A–B & D were repeated without adding in any biological cells.] Since there were no membrane capacitance, no capacitance and series resistance compensations were applied.*”

 And to Methods:

 “ *Similar to the no-cell experiments, since there were no cell membrane capacitance, no capacitance and series resistance compensations were applied.*”

 [1] Lei et al. 2020. Accounting for variability in ion current recordings using a mathematical model of artefacts in voltage-clamp experiments. *Phil. Trans. R. Soc. A*. 378:20190348. https://doi.org/10.1098/rsta.2019.0348

***The seal resistance values for the recordings shown in Figs. 2 and 3 are not given. Those values would allow the reader to interpret the amplitude of the leak current shown. This information is critical specially when comparing the results in Figure 3.II and Figure 3.III.***

 This is an interesting question as to whether we are able to accurately measure (or rather estimate) the seal resistance values. Whether we can safely use the definition of seal resistance is perhaps a better question. Seal resistance is defined as the inverse of the gradient of the I-V relationship, thus is measured (or estimated) for example using two points (i.e. two voltage steps) in the I-V relationship ideally when all ion channels are closed. In Figures 2 and 3, we can see that the I-V relationship of the leak current is non-linear, and that we cannot easily separate between the linear leak due to the imperfect seal (hence defining the seal resistance values) and the nonlinear time-dependent part of the leak current due to the crystals, hence it would be difficult and inaccurate to simply quote the seal resistance values for interpretation.

 We have added this explanation to the main text in Methods, reads as:

 “ *Note that although we use the seal resistance as a reference of the quality of the seal, we avoid directly quoting the values for overinterpretation. Seal resistance is defined as the inverse of the gradient of the (linear) I-V relationship, thus is usually estimated using two voltage steps in the I-V relationship, ideally when all ion channels are closed. However here we are examining a nonlinear time-dependent leak current, making direct interpretation of the seal resistance values inaccurate.*”

***Each of the I-Vs shown in Figures 2 and 3 appear to be derived from only one recording. Please provide data from multiple wells (Fig 2) and patches (Fig 3) and show means, standard errors (or standard deviations) and statistical significance. The conclusions would be supported by providing I-Vs derived from multiple observations not just from one.***

 Thanks for the suggestion. Multiple repeats of the experiments were included, and boxplots of the I-V curves are shown in all figures. The results were the same as the previously shown example, therefore we did not change the conclusion that we drew from the observations.

***The slope of the outward current in Fig.2A and 2B is steeper than the one shown in Fig 2C. It is assumed that no currents are going through the cell membrane on those recordings but this may not be the case. Is the block of hERG1a by E-4031 100%? There could also be chloride channels (Gill et al, 2006)1 that may be carrying the observed current. The conclusion that there is no current going through ion channels in those recordings would be strengthened if cesium was used instead of potassium (or using non-transfected CHO-K1 cells) and chloride channel blockers were added.***

 Thanks for the suggestion. Additional experiments was carried out with non-transfected CHO cells using a manual patch clamp system and the automated patch clamp platform with the same Ca-F containing solution, which is shown in new Figure 2C and D, showing the same nonlinear current as observed in Figure 2A and B. The difference between the slopes (Figure 2A-D and Figure 2E) was due to the ratio between the linear leak and the ‘extra’ time-dependent leak due to CaF2, as the no-cell experiment is expected to have a much larger linear leak. To confirm that they are the same type of time-dependent leak, a new Figure 3 is included, showing the time constants of the currents (for Figure 2A-D, Figure 2E and Measurement I) are the same.

***Regarding the results shown in Figure 3, the authors should compare their observations to those previously reported by Sachs and Qin (1993)* *2*** ***. The previous results showed ion-selective currents in the absence of CaF2 using a similar approach (glass pipette plus sylgard).***

 Thank you for the reference, indeed it is an interesting comparison. They have shown a nonlinear type of leak current when using 400mM NaCl for the pipette solution and 400mM arginine for the bath solution. Although for all other non-organic solution combinations they observed a ‘typical’ linear leak current as we did in our Measurement III. We have now included a mention of this paper at the start of the discussion:

 “ *[...]; observing a linear I-V relationship in Measurement III is indeed consistent with a previous study using Sylguard by Sachs and Qin (1993), whilst they also observed a nonlinear current in the presence of arginine.*”

***Is the linearity of the IV curve shown in Fig. 3.III due to the absence of fluoride or to the large drop in seal resistance? Please provide pre- and post-wash seal resistance values. Also multiple recordings (“patches”) would be recommended. Is the drop in resistance due to the loss of fluoride (and presumably CaF2 crystals)? Or to the pipette and Sylgard moving apart during the wash? Performing a wash with a fluoride-containing solution could answer this question.***

 This was a weakness of the original manuscript, the wash-in of a fresh solution for Measurement II could have disturbed the CaF2 crystal and created more large holes for a linear leak current in parallel with a remaining nonlinear current. However, upon repeating the Sylguard Meaurement III to improve the ‘ *n*’ for this revision we did observe cells where there was very little change in apparent seal resistance and we still saw the nonlinear leak disappear, as reflected in the new figures, pointing to the disappearance of the nonlinear current in the absence of fluoride rather than the addition of a much larger linear leak.

# Peer review: Reviewer response for version 1

**DOI:** 10.21956/wellcomeopenres.17512.r39338

**Published:** 2020-07-15

**Authors:** Marc Rogers

**Affiliations:** 1 Metrion Biosciences Limited, Cambridge, UK

Lei *et al.* expand on a historical automated patch clamp (APC) study of hERG current kinetics with a replicate APC study from a collaborator laboratory, to confirm the evidence for a nonlinear time- and voltage-dependent current component of unknown origin that would be problematic to subtract or remove using traditional pharmacological or biophysical techniques. In an effort to determine the basis of this non-linear current they emulate it using manual patch pipette recordings from an artificial silicone ’cell’, concluding that the combination of a high (internal) concentration of fluoride ions and a physiological concentration of (external) Ca2+ ions either side of a membrane seal produces a CaF crystal that can lead to non-linear current in APC recordings.

- Is the work clearly and accurately presented and does it cite the current literature?

Yes

- Is the study design appropriate and does the work have academic merit?

Partly – lack of cell-based data to back-up claims made using artificial silicone cell.

- Are sufficient details of methods and analysis provided to allow replication by others?

Yes

- If applicable, is the statistical analysis and its interpretation appropriate?

Partly – additional replicates, means and statistical variation data would be useful.

- Are all the source data underlying the results available to ensure full reproducibility?

Yes

- Are the conclusions drawn adequately supported by the results?

Partly - see below.

 Starting in the Abstract, the authors fail to adequately discriminate and make clear that their findings, and technical data, do not apply to all automated patch clamp platforms. It is neither fair nor accurate to paint all APC platforms with the same brush, as seems common in some academic papers and comments on this technology. Each APC platform is different, and their experience and main point about the combination of internal fluoride and high external divalent ‘seal enhancer’ being obligate to achieving gigaohm seals primarily applies to a single APC platform manufacturer.

 The second major issue is that the authors seem to suggest that a combination of high internal fluoride and external divalent cations is required to acquire gigaohn seals on APC platforms, and again this is inaccurate and may reflect their lack of experience with multiple platforms. It is true that some APC platforms may rely on high internal fluoride to achieve a high frequency of gigaohn seals, but this is neither obligate nor common these days, and it is actually contraindicated to combine high internal fluoride and elevated external divalents for many APC platforms.

 Also, the authors just tested a single combination of internal F and external Ca ^2+^ (Table 1), but should and could have looked at several different combinations and concentrations (as suggested in the Discussion) to determine the true source and magnitude of the CaF effect. In addition, many groups can achieve gigaseals without using >100 mM internal fluoride through a combination of optimised cell culture and experimental conditions, and biocompatible chip substrates. Thus, the statements in the Abstract that “ *With automated platforms it can be difficult to obtain such a high seal resistance between the intra- and extra-cellular solutions*” and “ *One suggested method to alleviate this problem is using an F  containing internal solution together with a Ca  containing external solution — so that a CaF  crystal forms when the two solutions meet which ‘plugs the holes’ to enhance the seal resistance*”, and the schematic and legend to Fig. 1B, are inaccurate and incorrect.

 As an APC user myself and a decades long patch clamper, I also have difficulty accepting the implication that the use of high internal fluoride and even low mM external divalents is a ‘trick’ solely employed for gigaseal APC recordings. I have lost track of the number of peer reviewed publications on voltage-gated Nav channels from leading academic groups, for example, that use this exact same recipe for their manual patch recordings, largely to ensure high resistance high fidelity recordings. Fig. 1A ignores this well-known tradition, assigning manual patch gigaseal resistances to the pipette glass-membrane tight seal alone. Thus, I would expect that a similar non-linear leak phenomenon would also be observable in many typical manual patch clamp recordings, but the authors notably did not run this experiment, and instead opted for a cell-free silicone-based biophysical manual patch pipette test. Thus, a claim or suggestion for ‘correction’ or close inspection of APC recordings employing a CaF effect would thus equally apply to a great number of past and future manual patch clamp datasets, but the authors do not include this possibility in their Abstract or Conclusions.

 The authors assume that all time- and voltage-dependent, exogenously expressed hERG channel current is removed in the presence of the pharmacological blocker, but do not provide evidence that this is the case under their recording conditions. By inference they suggest this is the case, and thus the similarity between the remaining non-linear outward leak current (Fig. 2A, B) and the open chip APC recording (Fig. 2C) is due to the CaF2 effect, rather than remaining hERG conductance.

 Similarly, the authors cite a reference on p4 that CHO cells have ‘little endogenous current’, but could have easily determined this empirically using wildtype or non-transfected cells and the same recording conditions and APC platforms used in the present study. Both sets of additional cell-based experiments would have bolstered their silicone ‘cell’ dataset, removing two possible related explanations for the APC cell non-linearity (leftover hERG and/or endogenous conductances, both of which would be expected to be time-dependent and non-linear at positive voltages), and strengthening their main claim about a non-linear CaF leak effect without relying on a simple ‘they look the same’ argument.

 Obviously there is a CaF-mediated non-linear biophysical phenomenom seen in the silicone-cell manual patch experiments, but acceptance of this manuscript and their (modified/clarified) claims about this affecting certain APC platform recordings requires actual cell-based data to compliment their historical APC datasets.

 Also, their Discussion suggestions at the bottom on p7 are somewhat out-of-date, as many of these options have already been explored by experienced APC users (e.g. reducing Fl and Ca concentrations, use of alternate cations).

 Finally, the language in the Conclusions needs to be re-worded to limit their claims to a certain type of APC platform (i.e. remove the plural to ‘APC platforms’). Also, the Conclusion stating that the CaF non-linear leak current is not due to endogenous (or non-blocked hERG) conductances is also not bourne out by the lack of manual patch clamp cell-based experimental data in this study, as outlined above.

Is the work clearly and accurately presented and does it cite the current literature?

Yes

If applicable, is the statistical analysis and its interpretation appropriate?

Partly

Are all the source data underlying the results available to ensure full reproducibility?

Yes

Is the study design appropriate and is the work technically sound?

Partly

Are the conclusions drawn adequately supported by the results?

No

Are sufficient details of methods and analysis provided to allow replication by others?

Yes

Reviewer Expertise:

Manual patch clamp, automated patch clamp, drug discovery screening, pharmacology, biophysics.

I confirm that I have read this submission and believe that I have an appropriate level of expertise to confirm that it is of an acceptable scientific standard, however I have significant reservations, as outlined above.

# Response

**Published:** 2021-10-26

**Authors:** Gary Mirams

**Affiliations:** University of Nottingham, UK

We thank the reviewer for their insightful review. This post relates to the changes introduced in Version 2 of the manuscript. The reviewer's comments are in bold and italic with our point-by-point responses below.

 To address the reviewer’s comments topic-by-topic, we have slightly re-ordered the reviewer’s comments here, but include them all.

***Starting in the Abstract, the authors fail to adequately discriminate and make clear that their findings, and technical data, do not apply to all automated patch clamp platforms. It is neither fair nor accurate to paint all APC platforms with the same brush, as seems common in some academic papers and comments on this technology. Each APC platform is different, and their experience and main point about the combination of internal fluoride and high external divalent ‘seal enhancer’ being obligate to achieving gigaohm seals primarily applies to a single APC platform manufacturer.***

 We completely agree that this is not a problem with APC platforms per-se. We were attempting a narrative style to start with where we first observed the problem, but agree that it perhaps confused the message. We have substantially re-worked the text to highlight that this is a problem with the solutions regardless of platform, rather than the platform.

***The second major issue is that the authors seem to suggest that a combination of high internal fluoride and external divalent cations is required to acquire gigaohn seals on APC platforms, and again this is inaccurate and may reflect their lack of experience with multiple platforms. It is true that some APC platforms may rely on high internal fluoride to achieve a high frequency of gigaohm seals, but this is neither obligate nor common these days, and it is actually contraindicated to combine high internal fluoride and elevated external divalents for many APC platforms. In addition, many groups can achieve gigaseals without using >100 mM internal fluoride through a combination of optimised cell culture and experimental conditions, and biocompatible chip substrates. Thus, the statements in the Abstract that “With automated platforms it can be difficult to obtain such a high seal resistance between the intra- and extra-cellular solutions” and “One suggested method to alleviate this problem is using an F  containing internal solution together with a Ca  containing external solution — so that a CaF  crystal forms when the two solutions meet which ‘plugs the holes’ to enhance the seal resistance”, and the schematic and legend to Fig. 1B, are inaccurate and incorrect.***

 We have substantially reworded the introductory text to remove the focus on APC platforms. Although not all APC experiments include the fluoride seal enhancer it has been very widely used; a recent cross-site and cross-platform comparison of ion channel screening for multiple voltage-gated cardiac ion channels was undertaken (Kramer et al., 2020). Whilst the data were anonymised, that publication details screening at 17 sites for hERG, Nav1.5 and CaV1.2 on 5 APC platforms, for a total of 39 assays being run (not all sites screened all channels). Non-proprietary internal solution data were available for 16/17 sites, 37/39 assays, 4/5 platforms. In our experiments we used 2.05mM [Ca ^2+^], 1mM [Mg ^2+^] and 100mM [F ^-^]. In the supplement of Kramer et al. (2020) we can see that 30/37 (81%) of cardiac ion channel screening assays used substantial amounts of fluoride (> 30mM) in the internal solution together with calcium in the external solution (at >1mM); and many used [F ^-^]>=120mM, whilst using similar amounts of Ca2+ to us (and most used Mg ^2+^ as well). Only 2 of 18 sites used no fluoride in any of their channel assays (but neither of these screened all three channels), and it was used on all 4 APC platforms in at least some of the channel assays.

***Also, the authors just tested a single combination of internal F and external Ca2+ (Table 1), but should and could have looked at several different combinations and concentrations (as suggested in the Discussion) to determine the true source and magnitude of the CaF effect.***

 In this short Research Note we are just hoping to draw attention to the problem, which we have not seen reported before, rather than propose a solution, so we have not examined the many factors which could influence the current size, but we have performed many more repeats of the original conditions to bolster the existing findings.

***The authors assume that all time- and voltage-dependent, exogenously expressed hERG channel current is removed in the presence of the pharmacological blocker, but do not provide evidence that this is the case under their recording conditions. By inference they suggest this is the case, and thus the similarity between the remaining non-linear outward leak current (Fig. 2A, B) and the open chip APC recording (Fig. 2C) is due to the CaF2 effect, rather than remaining hERG conductance. Similarly, the authors cite a reference on p4 that CHO cells have ‘little endogenous current’, but could have easily determined this empirically using wildtype or non-transfected cells and the same recording conditions and APC platforms used in the present study. Both sets of additional cell-based experiments would have bolstered their silicone ‘cell’ dataset, removing two possible related explanations for the APC cell non-linearity (leftover hERG and/or endogenous conductances, both of which would be expected to be time-dependent and non-linear at positive voltages), and strengthening their main claim about a non-linear CaF leak effect without relying on a simple ‘they look the same’ argument. [...] Obviously there is a CaF-mediated non-linear biophysical phenomenon seen in the silicone-cell manual patch experiments, but acceptance of this manuscript and their (modified/clarified) claims about this affecting certain APC platform recordings requires actual cell-based data to compliment their historical APC datasets. [...] Also, the Conclusion stating that the CaF non-linear leak current is not due to endogenous (or non-blocked hERG) conductances is also not bourne out by the lack of manual patch clamp cell-based experimental data in this study, as outlined above.***

 Thank you for the suggestion. Additional experiments were carried out with non-transfected CHO cells using both a manual patch clamp system and the automated patch clamp platform with the same Ca-F containing solution, which are shown as new panels in Figure 2C and D.

 The same nonlinear time-dependent current was observed even with non-transfected CHO cells, on both the automated and manual set ups, which confirms that the observed nonlinear current was not due to (potentially) remaining hERG conductance in Figure 2A and B.

***As an APC user myself and a decades long patch clamper, I also have difficulty accepting the implication that the use of high internal fluoride and even low mM external divalents is a ‘trick’ solely employed for gigaseal APC recordings. I have lost track of the number of peer reviewed publications on voltage-gated Nav channels from leading academic groups, for example, that use this exact same recipe for their manual patch recordings, largely to ensure high resistance high fidelity recordings. Fig. 1A ignores this well-known tradition, assigning manual patch gigaseal resistances to the pipette glass-membrane tight seal alone. Thus, I would expect that a similar non-linear leak phenomenon would also be observable in many typical manual patch clamp recordings, but the authors notably did not run this experiment, and instead opted for a cell-free silicone-based biophysical manual patch pipette test. Thus, a claim or suggestion for ‘correction’ or close inspection of APC recordings employing a CaF effect would thus equally apply to a great number of past and future manual patch clamp datasets, but the authors do not include this possibility in their Abstract or Conclusions.***

 As above, we have reworded the text to state that it is a problem with CaF _2_ but not automated patch clamp platforms themselves, and to highlight that manual experiments with the same solutions are subject to the same problems (as indeed our new manual experiments show). We agree that there are manual patch experiments in the literature that used Ca/F solutions. We have further added the following references in Introduction to emphasise this, reads as:

 “ *For instance, a F* ^*−*^ *containing internal solution together with a Ca* ^*2+*^ *containing external solution has been used with an early planar microfluidics setup (Kostyuk et al., 1975) and manual patching (Maltsev et al., 1997; Rugiero et al., 2003; Wang et al., 1996; Wendt et al., 1992), [...]*”

 We have also further noted in Dicussion that some manual patch studies may need to be re-examined because of this nonlinear time-dependent leak, reads as:

 “Some manual patch clamp findings in the literature may also need to be re-examined in light of these observations.”

***Also, their Discussion suggestions at the bottom on p7 are somewhat out-of-date, as many of these options have already been explored by experienced APC users (e.g. reducing Fl and Ca concentrations, use of alternate cations).***

 We could not find any reports of these currents in the literature, and therefore also haven’t seen any reports on attempts to minimise them. But we would be happy to reference any examples that you can point us to. 

***Finally, the language in the Conclusions needs to be re-worded to limit their claims to a certain type of APC platform (i.e. remove the plural to ‘APC platforms’).***

 As above, we have reworded the Conclusions to state that the observation of nonlinear leak is due to the use of Ca-F containing solutions rather than APC. It now reads as:

 “ *We recorded a nonlinear and time-dependent current in the presence of Ca* ^*2+*^ *and F* ^*−*^ *in patch clamp solutions (intended to form a CaF* *2* *seal enhancer).*”

# Peer review: Reviewer response for version 1

**DOI:** 10.21956/wellcomeopenres.17512.r39339

**Published:** 2020-07-14

**Authors:** Andrew Glazer

**Affiliations:** 1 Vanderbilt Center for Arrhythmia Research and Therapeutics, Department of Medicine, Vanderbilt University Medical Center, Nashville, TN, USA

Lei *et al.* describe a non-linear leak current that appears in the presence of fluoride, an ion used in automated patch clamp instruments to enhance seals. This unexpected leak current, not modeled by traditional leak current linear adjustment calculations, could interfere with the accurate characterization of ion channel properties on these instruments. Using manual patch clamp experiments with a silicone model of a cell, they reproduce the “extra” leak current and demonstrate that it disappears when the fluoride is washed out.

 The paper is clear and well-written, and describes an important finding that has implications for the growing field of automated patch clamp electrophysiology. The authors make the raw data and code available, and some of their observations were reproduced by 2 different laboratories/instruments.

 Major comments: The authors propose one solution to this problem, which is to do a full blocker-subtraction for all measurements, which could indeed help remove this “extra” leak current. However as the authors note, the properties of the seal might change over the course of the blocker addition (and some protocols are even longer to carry out than their staircase protocol). But what about actually removing the current with an internal solution exchange - another option the authors briefly mention in the discussion? Could they generate the initial tight seal on the SyncroPatch using an fluoride-containing internal solution and calcium-containing external solution, then do an internal solution exchange that removes the fluoride? Would the seals remain strong and the extra leak current go away, as they saw with Measurement 3 in manual patch clamp? If successful, this could demonstrate a relatively easy solution to this problem on the SyncroPatch. The authors briefly propose this experiment in the discussion as a future direction but it would be a nice addition to this paper to demonstrate it.

 Please present multiple replicate cells for the I-V curves and show means and standard errors.

 Minor comments:

 The staircase protocol is a nice alternative to the standard hERG protocols and is described in the methods. But it would be helpful to mention it briefly in the Introduction and give a citation to the papers by the same authors that developed it to avoid confusing readers who might be expecting standard hERG protocols.

 The paper is best read linearly including fully reading the Methods to understand the logic of the silicone and the experimental measurements. However I worry readers might skip the methods and jump to the results where there is not much explanation/motivation for the 3 measurements. This could be solved by adding more motivating text to the start of the results and to the start of each experimental measurement paragraph. For example, the first two paragraph of the methods could be moved to become the first two paragraphs of the results. And a sentence or two could be added to the start of each measurement paragraph in the results to describe the aim/goal of the measurement, for readers who didn’t read or skimmed the Methods.

 Comparing 3-II to 3-III, although the leak current now appears to be “normal” (extra leak current removed), is the magnitude of the leak current higher after the fluoride solution is washed out? Could the authors present data on the resistances/leak current magnitudes before and after fluoride washout? If there is a less tight seal, would this prevent the success of the internal solution exchange strategy on the SyncroPatch because the seals would decrease following washout?

 After some confusion, I realized that X's in the the I-V curves in figures 2+3 each show 1 cell from the staircase protocol, with multiple X's at the same voltage. Please clarify this in the legend.

 The author’s charged plug model for the leak current is a bit speculative - but it is caveated as a “tentative” model/hypothesis.

Is the work clearly and accurately presented and does it cite the current literature?

Yes

If applicable, is the statistical analysis and its interpretation appropriate?

Yes

Are all the source data underlying the results available to ensure full reproducibility?

Yes

Is the study design appropriate and is the work technically sound?

Yes

Are the conclusions drawn adequately supported by the results?

Partly

Are sufficient details of methods and analysis provided to allow replication by others?

Yes

Reviewer Expertise:

Automated patch clamping.

I confirm that I have read this submission and believe that I have an appropriate level of expertise to confirm that it is of an acceptable scientific standard, however I have significant reservations, as outlined above.

# Response

**Published:** 2021-10-26

**Authors:** Gary Mirams

**Affiliations:** University of Nottingham, UK

We thank the reviewer for their thoughtful review. This post relates to the changes introduced in Version 2 of the manuscript. The reviewer's comments are in bold and italic with our point-by-point responses below.

***Major comments:***

***The authors propose one solution to this problem, which is to do a full blocker-subtraction for all measurements, which could indeed help remove this “extra” leak current. However as the authors note, the properties of the seal might change over the course of the blocker addition (and some protocols are even longer to carry out than their staircase protocol). But what about actually removing the current with an internal solution exchange - another option the authors briefly mention in the discussion? Could they generate the initial tight seal on the SyncroPatch using an fluoride-containing internal solution and calcium-containing external solution, then do an internal solution exchange that removes the fluoride? Would the seals remain strong and the extra leak current go away, as they saw with Measurement 3 in manual patch clamp? If successful, this could demonstrate a relatively easy solution to this problem on the SyncroPatch. The authors briefly propose this experiment in the discussion as a future direction but it would be a nice addition to this paper to demonstrate it.***

 Thank you for the suggestion. Measurement III indeed suggested a potential method for removing the nonlinear time-dependent leak current. In this short ‘Research Note’ we are just hoping to draw attention to the problem rather than propose a particular solution - which may depend on the precise solutions being used, the cell types and the particular manual or automated patch platforms that are in use.

***Please present multiple replicate cells for the I-V curves and show means and standard errors.***

 Thank you for the suggestion, more repeats of the experiments are shown in all figures for the I-V curves.

***Minor comments:***

***The staircase protocol is a nice alternative to the standard hERG protocols and is described in the methods. But it would be helpful to mention it briefly in the Introduction and give a citation to the papers by the same authors that developed it to avoid confusing readers who might be expecting standard hERG protocols.***

 Thanks for the suggestion, it is now introduced in Introduction: “ *The measurements were done under a voltage-clamp protocol used in Lei et al. (2019a), known as the “staircase protocol” (see top panel of Figure 2).*”

***The paper is best read linearly including fully reading the Methods to understand the logic of the silicone and the experimental measurements. However I worry readers might skip the methods and jump to the results where there is not much explanation/motivation for the 3 measurements. This could be solved by adding more motivating text to the start of the results and to the start of each experimental measurement paragraph. For example, the first two paragraph of the methods could be moved to become the first two paragraphs of the results. And a sentence or two could be added to the start of each measurement paragraph in the results to describe the aim/goal of the measurement, for readers who didn’t read or skimmed the Methods.***

 Thanks for the suggestion, indeed the paper was intended to be read linearly, as we wanted to give the logical flow of our observations and followed up experiments. We have now added more motivating text to the start of the results and to the start of each experimental measurement paragraph in the Results section.

***Comparing 3-II to 3-III, although the leak current now appears to be “normal” (extra leak current removed), is the magnitude of the leak current higher after the fluoride solution is washed out? Could the authors present data on the resistances/leak current magnitudes before and after fluoride washout? If there is a less tight seal, would this prevent the success of the internal solution exchange strategy on the SyncroPatch because the seals would decrease following washout?***

 The reviewer is correct, here the lower resistance suggests a larger leak which may be going ‘around’ rather than ‘through’ the crystal. We have now repeated the experiments (Measurements II and III), out of the four repeats, only two of them stayed approximately 'the same size' after the fluoride solution was washed out (as shown in the new Figure 4). We expect that there is still a potential to use the internal solution exchange strategy on the SyncroPatch but it would require further exploration and testing, hence we leave this as a potential future study.

***After some confusion, I realized that X's in the I-V curves in figures 2+3 each show 1 cell from the staircase protocol, with multiple X's at the same voltage. Please clarify this in the legend.***

 New experiments for multiple cells/measurements have now been repeated and included in the I-V curves in all figures. We also explain the I-V curves contain more than one data point per cell at the same voltage in the figure caption, reads as

 “ *Note that each boxplot can contain more than one data point per cell due to the repeat of the voltage step in the protocol.*”

***The author’s charged plug model for the leak current is a bit speculative - but it is caveated as a “tentative” model/hypothesis.***

 Thank you for the comment, indeed we do not claim it was the conclusion drawn from the data presented in this manuscript. It was just a hypothesis that we made after observing the phenomenon.

# Peer review: Reviewer response for version 1

**DOI:** 10.21956/wellcomeopenres.17512.r39341

**Published:** 2020-07-13

**Authors:** Gildas Loussouarn

**Affiliations:** 1 CNRS, INSERM,the thorax institute, University of Nantes, Nantes, France

In the present manuscript, Lei and collaborators report the observation of a rectifying and time-dependent current, after the generation of Giga-seals enhanced by CaF crystals, a procedure that is unavoidable for several automated patch-clamp platforms using the planar patch configuration. This observation is of interest since such current, if not removed, may pollute the current under study.

 The authors replaced a living cell by a drop of Sylgard in a conventional patch-clamp set-up to mimic an imperfect seal. Using this original procedure, they could observe a CaF-induced leak current, in absence of any plasma membrane endogenous current. However, some complementary experiments should be added to give more insights on the CaF-induced current and avoid any over-interpretation of the data.

1. The panels (A) and (B) of Figure 2 show strikingly similar recordings from two different laboratories using the same solutions in the same type of Syncropatch machine. Average of currents, at least for the I/V curve would be more convincing than comparing two representative cells. 
2. Panel (C) is quite different to panel (A) and (B), with similar time dependent currents generated by a large depolarization (-80 to +40 mV) but not by smaller depolarization (0 to +40 mV). In Syncropatch systems, to avoid over–compensation and current oscillations that can disrupt the seal, Rseries feedback compensation of the recorded current is applied with a slow time constant. Given the high current amplitude (several nA), is it possible that the time-dependent current observed in panel C is due to slowly developing feedback compensation? 
3. Figure 3, when switching internal and external solutions, it would be more relevant to also invert the polarity of the voltage protocol and to directly compare the superimposed currents. Moreover, as in point 1, average of currents, at least for the I/V curve would be more convincing. 
4. Experiments in Figure 2 and 3 are quite different, with, in Figure 2, an automated patch-clamp system and a real cell whereas in Figure 3, a conventional patch-clamp system and an artificial cell. There may some other models without endogenous current, that can be used in automated patch-clamp, such as giant unilamellar vesicles ^1^. Another option would be to test if the currents observed in Figure 2 and 3 are of the same nature, by replacing internal K ^+^ and/or Na ^+^ by the organic cation N-methyl-D-glucamine (NMDG) in the intracellular medium (be very careful if you need to use HF to prepare this solution, HF is a very corrosive and extremely toxic acid) or by replacing Cl ^-^ by gluconate in the extracellular medium. Indeed, since the time-dependent current is an outward current, it may be carried by cations diffusing from the intracellular medium to the extracellular medium and/or by anions diffusing from the extracellular medium to the intracellular medium. Fluoride diffusion is unlikely the basis of the observed current, as suggested in the discussion: “ *Furthermore, F ^−^ has a higher mobility than Ca ^2+^, so F ^−^ may preferentially move out through the imperfect seal and form crystals with Ca ^2+^ on the Ca ^2+^-side of the membrane. This hypothesis is consistent with the direction of the ‘extra’ current in the leak in both Measurements I and II.*” 

Minor points

1. In ‘Data Analysis’, “ *E _leak_ is directly calculated from equation (1) using one of the voltage step*”: it would be interesting to indicate if E _leak _calculations gives values close to zero. 
2. In the results, sentence “ *The measured leak current was time-dependent when it was held at a constant voltage, and it showed a noticeable outward (positive) time-dependent current during an increase of voltage from zero to 40 mV*“. I don’t understand the difference between the two described time-dependence. I think the sentence could be simpler. 
3. In the discussion: it is proposed that when fluoride is removed, CaF crystals remain, suggesting that both CaF and F ^-^ need to be present to observe the time dependent and rectifying current. The fact that seals quality deteriorates upon F ^-^ removal suggest that CaF crystals disappear, so the loss of the outwardly rectifying current may be due to the loss of crystals, and for instance, the loss of a rectifying current carried by intracellular cations in the crystal lattice, as suggested in “major point 4”. There is no strong argument for the direct presence of fluoride as a cause of the outwardly rectifying current. 
4. Authors should indicate other issues regarding the use of intracellular fluoride and extracellular calcium as a seal enhancer. Fluoride is (i) a phosphatase inhibitor and, obviously here, (ii) a calcium chelator preventing the use of calcium containing intracellular solutions. Using extracellular calcium at higher concentration than the physiological range alters channel gating ^2^, at least partly due to membrane charge screening ^3^.

Is the work clearly and accurately presented and does it cite the current literature?

Yes

If applicable, is the statistical analysis and its interpretation appropriate?

Partly

Are all the source data underlying the results available to ensure full reproducibility?

Yes

Is the study design appropriate and is the work technically sound?

Partly

Are the conclusions drawn adequately supported by the results?

Partly

Are sufficient details of methods and analysis provided to allow replication by others?

Yes

Reviewer Expertise:

Electrophysiology, patch-clamp.

I confirm that I have read this submission and believe that I have an appropriate level of expertise to confirm that it is of an acceptable scientific standard, however I have significant reservations, as outlined above.

## References

[1] : Human TRPA1 is an inherently mechanosensitive bilayer-gated ion channel. *bioRxiv* .2020; 10.1101/2020.03.05.979252 10.1101/2020.03.05.979252 32717533

[2] : Voltage-dependent blockade of HERG channels expressed in Xenopus oocytes by external Ca2+ and Mg2+. *J Physiol* .1998;507 ( Pt 3): 10.1111/j.1469-7793.1998.631bs.x 631-8 10.1111/j.1469-7793.1998.631bs.x 9508824PMC2230832

[3] : Divalent ions and the surface potential of charged phospholipid membranes. *J Gen Physiol* .1971;58(6) : 10.1085/jgp.58.6.667 667-87 10.1085/jgp.58.6.667 5120393PMC2226047

# Response

**Published:** 2021-10-26

**Authors:** Gary Mirams

**Affiliations:** University of Nottingham, UK

We thank the reviewer for the useful and insightful comments. Bold and italic below are the reviewer's comments with our point-by-point response below to describe updates in the revised version of the manuscript.

***> The panels (A) and (B) of Figure 2 show strikingly similar recordings from two different laboratories using the same solutions in the same type of Syncropatch machine. Average of currents, at least for the I/V curve would be more convincing than comparing two representative cells.***

 Thanks for the suggestion. More repeats of the experiments were performed and included, and boxplots of the resulting I-V curves are shown in Figure 2. Further analysis of the time constants of the currents is now also included in Figure 3B.

***> Panel (C) is quite different to panel (A) and (B), with similar time dependent currents generated by a large depolarization (-80 to +40 mV) but not by smaller depolarization (0 to +40 mV). In Syncropatch systems, to avoid over–compensation and current oscillations that can disrupt the seal, Rseries feedback compensation of the recorded current is applied with a slow time constant. Given the high current amplitude (several nA), is it possible that the time-dependent current observed in panel C is due to slowly developing feedback compensation?***

 Indeed the compensation may affect the shapes of the observed current, but since it couldn’t estimate the membrane capacitance (as there was no cell), it would not be able to perform Rseries compensation. This has now been explained in the main text. Furthermore, we have conducted additional experiments with non-transfected CHO cells, and repeated these using both the conventional patch-clamp system and the automated patch-clamp system with the Ca-F containing solutions, with results shown in the new Figure 2C and D panels. We observed the same type of nonlinear current as those observed in Figure 2A and B and Measurement I.

***> Figure 3, when switching internal and external solutions, it would be more relevant to also invert the polarity of the voltage protocol and to directly compare the superimposed currents.***

 We thank the reviewer for the suggestion. However, since we do not know exactly the electrochemical properties of the nonlinear time-dependent part of the current, for example its reversal potential etc., we do not attempt to invert the polarity of the voltage protocol as it may not give an easily interpretable direct comparison.

***> Moreover, as in point 1, average of currents, at least for the I/V curve would be more convincing.***

 More repeats of the experiments were undertaken and included for the I-V curves shown as boxplots.

***> Experiments in Figure 2 and 3 are quite different, with, in Figure 2, an automated patch-clamp system and a real cell whereas in Figure 3, a conventional patch-clamp system and an artificial cell. There may some other models without endogenous current, that can be used in automated patch-clamp, such as giant unilamellar vesicles. Another option would be to test if the currents observed in Figure 2 and 3 are of the same nature, by replacing internal K+ and/or Na+ by the organic cation N-methyl-D-glucamine (NMDG) in the intracellular medium (be very careful if you need to use HF to prepare this solution, HF is a very corrosive and extremely toxic acid) or by replacing Cl- by gluconate in the extracellular medium.***

 We have included extra experiments with non-transfected CHO cells repeated using both the conventional patch-clamp system and the automated patch-clamp system with the Ca-F containing solutions. We observed the same type of nonlinear current as those observed in Figure 2A and B and Measurement I. We further analyzed the time constant of the observed current, as shown in new Figure 3. In particular Figure 3B shows that all the time dependent part of the observed current in all experiments A-E and Measurement I are the same.

***> Indeed, since the time-dependent current is an outward current, it may be carried by cations diffusing from the intracellular medium to the extracellular medium and/or by anions diffusing from the extracellular medium to the intracellular medium. Fluoride diffusion is unlikely the basis of the observed current, as suggested in the discussion: “Furthermore, F− has a higher mobility than Ca2+, so F− may preferentially move out through the imperfect seal and form crystals with Ca2+ on the Ca2+-side of the membrane. This hypothesis is consistent with the direction of the ‘extra’ current in the leak in both Measurements I and II.”***

 Thank you for this comment, you are correct that the nonlinear outward current would be either cations moving from the intracellular medium to the extracellular medium and/or anions moving from the extracellular to the intracellular. The quoted text from the main text ” *Furthermore, F- has a higher mobility [...]*” simply refers to where the crystal would form, which would happen before the measurements were done, hence we did not mean to suggest that the observed current *was* fluoride moving from the intracellular medium to the extracellular medium, just that it was caused by it. We agree that the text was confusing, we’ve replaced it with:

 “ *Our leading conjecture is that the nonlinear time-dependent leak current is ordinary linear leak through imperfect seal with a conductance that changes nonlinearly and time-dependently with voltage. We speculate that the reason for this ‘extra’ current could be defects in the CaF2 crystals: F− has a higher mobility than Ca2+, so F− may preferentially move back into the cell from imperfections in the crystal when voltage is high. The extra defects in the crystal that result (sites missing fluoride, Huisinga, 1999) could allow a larger non-selective leak, forming the measured current. This process would reverse when voltage is low, with fluoride returning to ‘plug the holes’ in the crystal and reducing leak conductance, giving the observed asymmetry in leak current. This is a similar concept to e.g. positively charged polyamines causing block of inward rectifier potassium channels at positive potentials (Fakler et al., 1995). That is, neither fluoride nor the polyamines contribute strongly to a measured current but are the cause of its block. This hypothesis is consistent with the direction of the ‘extra’ current in the leak in both Measurements I and II.*”

**> *Minor points***

***In ‘Data Analysis’, “Eleak is directly calculated from equation (1) using one of the voltage step”: it would be interesting to indicate if Eleak calculations gives values close to zero.***

 The estimated Eleak values across all recordings are 8.14 ± 3.38 mV (mean ± SEM) which is relatively close to zero, but we do observe a fair amount of variation in Eleak. We have included this in the main text.

***> In the results, sentence “The measured leak current was time-dependent when it was held at a constant voltage, and it showed a noticeable outward (positive) time-dependent current during an increase of voltage from zero to 40 mV“. I don’t understand the difference between the two described time-dependence. I think the sentence could be simpler.***

 We have reworded the sentence, which reads as:

 “ *The measured leak current was time-dependent when it was held at a constant voltage, this is most noticeable for the outward (positive) current during an increase of voltage from 0 to +40 mV.*”

***> In the discussion: it is proposed that when fluoride is removed, CaF crystals remain, suggesting that both CaF and F- need to be present to observe the time dependent and rectifying current. The fact that seals quality deteriorates upon F- removal suggest that CaF crystals disappear, so the loss of the outwardly rectifying current may be due to the loss of crystals, and for instance, the loss of a rectifying current carried by intracellular cations in the crystal lattice, as suggested in “major point 4”. There is no strong argument for the direct presence of fluoride as a cause of the outwardly rectifying current.***

 The reviewer is correct that in the previous Measurement III, the lower resistance suggests a larger leak which may be going ‘around’ rather than ‘through’ the crystal or even be due to the loss of the crystal. Additional experiments were performed for Measurements II and III. In two of the four repeats, the seal quality (the leak current size) stayed the same whilst the nonlinear time-dependent current still disappeared after the fluoride solution was washed out. This is shown in Figure 4 (a new version of Figure 3), supporting the proposed hypothesis.

***> Authors should indicate other issues regarding the use of intracellular fluoride and extracellular calcium as a seal enhancer. Fluoride is (i) a phosphatase inhibitor and, obviously here, (ii) a calcium chelator preventing the use of calcium containing intracellular solutions. Using extracellular calcium at higher concentration than the physiological range alters channel gating, at least partly due to membrane charge screening.***

 Thank you for the suggestion. The following text has now been added to the end of Discussion:

 “ *In addition to the extra nonlinear time-dependent leak current, the use of intracellular fluoride and extracellular calcium as a seal enhancer could also give rise to other problems. Using intracellular fluoride, as a calcium chelator, prevents the use of calcium containing intracellular solutions, and fluoride is also a phosphatase inhibitor (Guranowski 1990). Using extracellular calcium at higher concentrations than the physiological range can also alter channel gating (Ho et al., 1998), at least partly due to membrane charge screening (McLaughlin et al., 1971).*”

 Guranowski A. Fluoride is a strong and specific inhibitor of (asymmetrical) Ap4A hydrolases. *FEBS letters*. 1990; 262(2):205-8.

 Ho WK, Kim I, Lee CO, Earm YE. Voltage-dependent blockade of HERG channels expressed in Xenopus oocytes by external Ca2+ and Mg2+. *J Physiol.* 1998; 507 (Pt 3): 631-8

 McLaughlin SG, Szabo G, Eisenman G. Divalent ions and the surface potential of charged phospholipid membranes. *J Gen Physiol*. 1971; 58 (6): 667-87
